# Global Demethylation of Rat Chondrosarcoma Cells after Treatment with 5-Aza-2′-Deoxycytidine Results in Increased Tumorigenicity

**DOI:** 10.1371/journal.pone.0008340

**Published:** 2009-12-17

**Authors:** Christopher A. Hamm, Hehuang Xie, Fabricio F. Costa, Elio F. Vanin, Elisabeth A. Seftor, Simone T. Sredni, Jared Bischof, Deli Wang, Maria F. Bonaldo, Mary J. C. Hendrix, Marcelo B. Soares

**Affiliations:** 1 Interdisciplinary Graduate Program in Genetics, University of Iowa, Iowa City, Iowa, United States of America; 2 Cancer Biology and Epigenomics Program, Falk Brain Tumor Center, Children's Memorial Research Center, Chicago, Illinois, United States of America; 3 Robert H. Lurie Comprehensive Cancer Center, Northwestern University's Feinberg School of Medicine, Chicago, Illinois, United States of America; 4 Biostatistics Research Core, Children's Memorial Research Center, Northwestern University's Feinberg School of Medicine, Chicago, Illinois, United states of America; Institute of Genetics and Molecular and Cellular Biology, France

## Abstract

Abnormal patterns of DNA methylation are observed in several types of human cancer. While localized DNA methylation of CpG islands has been associated with gene silencing, the effect that genome-wide loss of methylation has on tumorigenesis is not completely known. To examine its effect on tumorigenesis, we induced DNA demethylation in a rat model of human chondrosarcoma using 5-aza-2-deoxycytidine. Rat specific pyrosequencing assays were utilized to assess the methylation levels in both LINEs and satellite DNA sequences following 5-aza-2-deoxycytidine treatment. Loss of DNA methylation was accompanied by an increase in invasiveness of the rat chondrosarcoma cells, *in vitro*, as well as by an increase in tumor growth *in vivo*. Subsequent microarray analysis provided insight into the gene expression changes that result from 5-aza-2-deoxycytidine induced DNA demethylation. In particular, two genes that may function in tumorigenesis, sox-2 and midkine, were expressed at low levels in control cells but upon 5-aza-2-deoxycytidine treatment these genes became overexpressed. Promoter region DNA analysis revealed that these genes were methylated in control cells but became demethylated following 5-aza-2-deoxycytidine treatment. Following withdrawal of 5-aza-2-deoxycytidine, the rat chondrosarcoma cells reestablished global DNA methylation levels that were comparable to that of control cells. Concurrently, invasiveness of the rat chondrosarcoma cells, *in vitro*, decreased to a level indistinguishable to that of control cells. Taken together these experiments demonstrate that global DNA hypomethylation induced by 5-aza-2-deoxycytidine may promote specific aspects of tumorigenesis in rat chondrosarcoma cells.

## Introduction

Aberrant DNA methylation is thought to play an integral role in the complex process of tumorigenesis [1]. Abnormal hypermethylation may result in the silencing of genes that are members of pathways ranging from cell division to tumor suppression. Reintroducing the expression of abnormally silenced genes may restore control of these various signaling and regulatory pathways.

Current epigenetic therapies are aimed at bringing on hypomethylation with the goal of reverting hypermethylation-induced gene silencing [2]. One such therapeutic agent is 5-Aza-2-deoxycytidine, which is a deoxycytidine analog that becomes incorporated into DNA and inhibits the activity of DNA methyltransferases [3], [4]. The incorporation of 5-Aza-2-deoxycytidine and subsequent inhibition of DNA methyltransferases results in reduced levels of DNA methylation [5], [6]. 5-Aza-2-deoxycytidine has been shown to have clinical benefits in the treatment of myelodysplastic syndrome and it has also been shown to be effective in the treatment of other myeloid malignancies [7].

Despite the potential benefits of 5-aza-2-deoxycytidine, the complete ramifications of treating tumor cells with a global DNA hypomethylating agent are unknown. The phenomenon of global DNA hypomethylation has been observed in several types of cancer [8], and DNA hypomethylation has also been associated with tumor aggressiveness [9]. Evidence also suggests that DNA hypomethylation may play a causal role in tumorigenesis [10], [11].

To study the impact of global DNA hypomethylation on the behavior of tumor cells we treated swarm rat chondrosarcoma (SRC) cells with 5-aza-2-deoxycytidine and monitored its effect both *in vitro* and *in vivo*. We selected the SRC tumor model based on its extensive characterization and the ability of the SRC cells to be grown and studied both *in vitro* and *in vivo* [12], [13], [14], [15], [16].

SRC cells were treated *in vitro* with a low dose of 5-Aza-2-deoxycytidine for 30 days to induce genome-wide hypomethylation and their level of methylation was assessed using rat specific pyrosequencing assays. Treatment with 5-Aza-2-deoxycytidine led to demethylation of both LINE and microsatellite regions throughout the genome. The effects of long-term exposure to epigenetic agents are not completely known, and a potential concern is that such treatment may lead to the expression of genes that are normally epigenetically silenced. In addition, it may cause illegitimate transcription events [17], [18]. Indeed, invasion assays performed with treated and untreated SRC cells indicated that loss of methylation is accompanied by an increase in invasiveness. Furthermore, microarray analysis revealed that 5-Aza-2-deoxycytidine treatment leads to alterations in expression of several developmentally regulated genes.

Based on their differential expression, two of these genes, midkine and sox-2, were selected for additional expression and epigenetic analyses. Midkine, a growth factor [19], and sox-2, a pluripotent transcription factor [20], are expressed at higher levels following 5-Aza-2-deoxycytidine treatment. Treatment with 5-Aza-2-deoxycytidine leads to loss of methylation in the promoter regions of both midkine and sox-2 genes, thus suggesting that methylation may play a role in the transcriptional regulation of these genes.

Since 5-Aza-2-deoxycytidine-induced hypomethylation resulted in several phenotypic changes in the SRC cells *in vitro*, we wanted to determine if the treatment would affect cell growth *in vivo*. Following subcutaneous transplantation, the 5-Aza-2-deoxycytidine treated SRC cells formed larger tumors than the corresponding untreated SRC cells. Methylation and expression analyses of the *in vivo* SRC cells revealed that the effect of 5-Aza-2-deoxycytidine could be observed for at least 60 days following treatment discontinuation.

Altogether the *in vivo* and *in vitro* results suggest that induction of genome-wide hypomethylation by 5-Aza-2-deoxycytidine results in an increase in the tumorigenicity of the SRC cells. The SRC experiments also highlight the importance that epigenetic modifications may have in cancer and suggest that DNA hypomethylation may have a functional role in tumor progression.

## Materials and Methods

### Ethics Statement

All animals were handled in strict accordance with good animal practice as defined by the relevant national and/or local animal welfare bodies, and all animal work was approved by the Institutional Animal Care and Use Committee (Children's Memorial Research Center; protocol IACUC #2006-30).

### Establishment of a Bioluminescent Rat Chondrosarcoma Cell Line

A Murine Stem Cell Virus-Luciferase-Internal ribosomal entry site-Hygromycin (MSCV-Luc-I-Hygro) retroviral vector was prepared by transfecting 293T cells with three plasmids; pMSCV-Luc-I-Hyrgo (which encodes the Luciferase and hygromycin phosphotransferase), pEQ-Pam3(-E) (which encodes retroviral gag and pol) and pSRα-G (which encodes glycoprotein G from Vesicular Stomatitis Virus) [21]. Forty-eight hours post-transfection media containing retroviral vector was collected, aliquoted, frozen, and stored at −80°C. This vector was then used to transduce the Swarm rat chondrosarcoma cell line (SRC-LTC (Long Term Culture) [13], [obtained from Jeff W. Stevens, University of Iowa]), in the presence of 5 µg/ml polybrene on three successive days allowing the cells to recover in the media generally used overnight. Transduced cells were selected by incubation with hygromycin B (Sigma-Aldrich, St. Louis, MO) at a concentration of 500 µg/ml for 14 days. Once the hygromycin resistant population was established, the cells were maintained in media containing 500 µg/ml of hygromycin B. The newly established rat chondrosarcoma cell line was named SRC-MSCV3-LTC.

### Cell Culture Conditions and 5-Aza-2-Deoxycytidine Treatment

SRC-MSCV3-LTC cells were cultured in DMEM high glucose (4.5 g glucose/ml) supplemented with 10% FBS and Penicillin/Streptomycin. Cells were plated at 2.5.×10^4^ cells with 6 ml of media in a 25 cm^2^ T flask. Cells were grown until they became 80–90% confluent (6 days), and at this time the cells were trypsinized/split and plated as described. For the 5-Aza-2-deoxycytidine treatment, the media was supplemented with 0.1 uM 5-Aza-2-deoxycytidine on the day that the cells were passaged. Cell viability was assessed 72 hours and 144 hours following 5-Aza-2-deoxycytidine treatment (0.1, 0.3, 1.0, 3.0, 10, 30, 100, and 300 µM). For the 72-hour treatment, the cells were treated with 5-Aza-2-deoxycytidine at day 0 and 72 hours later the viability was examined (Figure S1A). For the 144-hour treatment, cells were treated with 5-Aza-2-deoxycytidine every 24 hours without changing the media as previously described [22], and 144 hours after the initial treatment the viability was examined (Figure S1B). Viability was determined using the Guava EasyCyte Mini Flow Cytometry System, and the Guava ViaCount Reagent (Millipore; cat no. 4000-0040).

The Swarm rat chondrosarcoma line SRC-MSCV3-LTC, was treated with 0.1 uM 5-Aza-2-deoxycytidine for 5 passages (30 days). Control cells were grown for 5 passages without 5-Aza-2-deoxycytidine. After 5 passages, cells were either frozen for subsequent DNA and RNA analysis, or they were passaged for five additional passages (30 days) without any drug treatment after which they were frozen for future analysis.

For *in vivo* experiments, cells were grown *in vitro* for 5 passages with or without 5-Aza-2-deoxycytidine. For the treated cells, the 5-Aza-2-deoxycytidine treatment was removed on the day of the injection and the cells did not receive further 5-Aza-2-deoxycytidine treatment.

### Tumor Inductions

Following growth for 5 passages, cells were injected subcutaneously into the lower lumbar region of 4 week old nude mice(Males; Charles River, Strain code: 088). The SRC cells were grown until they were 80% confluent, the cells were then washed with PBS, and then cells were removed from the plate using TrypLE Express (GIBCO cat#: 12605-010) according to manufactures instructions. Following removal of SRC cells from plates, the cells were washed with PBS, centrifuged, and resuspended in PBS. Either 1×10^6^, 5×10^6^, or 10×10^6^ cells were injected subcutaneously. For each experiment one animal was injected with untreated control SRC cells, and one animal was injected with 5-Aza-2-deoxycytidine treated cells. The animals did not receive any dose of 5-Aza-2-deoxycytidine.

Following the injection, the animals were monitored twice weekly for 60 days. After 60 days the animals were euthanized by CO_2_ gas inhalation followed by cervical dislocation. Immediately following euthanization, tumors and other tissues were frozen in liquid nitrogen or placed in paraformaldehyde for histology.

### *In Vivo* Imaging

All in vivo imaging was performed with the Xenogen IVIS 200 imaging system. Ten minutes prior to imaging, D-luciferin (150 mg/kg of body weight) was injected into the intraperitoneal cavity of the mice. During the image acquisition animals were anesthetized with isoflurane inhalation at 1 to 2%.

### Primer Design and Pyrosequencing

Rat genome sequence (rn4/version 3.4, Nov. 2004) and the annotation for repetitive elements were obtained from the UCSC Genome Database. Based on genomic co-ordinates of LINE elements provided by the UCSC database, 899,092 LINE sequences were extracted and subjected to *in silico* bisulfite treatment. 8,460 L1 elements with length over 6000 bp were identified and used for alignment to generate LINE nucleotide base matrix. A region within L1 elements with dense CpG dinucleotides was selected for PCR primer design. An electronic PCR was performed with the novel primers designed for rat LINEs. 827 LINE elements in the rat genome would be targeted in PCR reactions with the primer set designed. With two sequencing primers, a total number of 7 CpG dinucleotides were sequenced for each LINE element targeted. The global methylation data generated was derived from a minimum number of 5,700 CpG dinucleotides in LINE elements. A similar approach was taken to design novel primers for rat satellite repeats. Primers targeting a minimum number of 137 distinct Satellite I elements and five distinct Satellite II elements were designed. For each Satellite element targeted, the methylation profiles were determined for three CpG dinucleotides. Primer sequence and reaction conditions are available in Table S1.

### Microarray

Microarray analysis was used to examine the gene expression profiles of the SRC cells (+) or (−)5-Aza-2-deoxycytidine treatment. Microarray was carried out using the NimbleGen microarray service. The *Rattus norvegicus* 1-plex array (14 probes/target; 26739 genes; cat#: A6184-00-01) was used for each hybridization. Two hybridizations were performed on 5-Aza-2-deoxycytidine treated SRC cells and three hybridizations were performed on untreated control SRC cells. Data were processed and displayed using Genespring software (Agilent Technologies). Genespring was used to identify differentially expressed genes that had a 5-fold difference between the 5-Aza-2-deoxycytidine treated samples and the untreated control samples. Additionally, Genespring was used to create a gene tree (Pearson coefficient) to graphically represent the data.

The list of differentially expressed genes was analyzed using a pathway-mapping program (Ingenuity Pathway Analysis version 7.0). Ingenuity was used to sort the list of differentially expressed genes (977genes) based on their role in cellular function and disease. Ingenuity identified 135 cancer related genes. For the heat map, the cancer gene list was further filtered by requiring a minimum expression level of at least 1,000 relative fluorescence units in at least 2 different hybridizations.

Genespring was used for hierarchical clustering, to create a gene tree (Pearson coefficient), and to generate the heat map used to graphically represent the data.

The list of genes with a 5-fold difference (977genes) was also analyzed using GeneGo to identify pathways that were altered following 5-Aza-2-deoxycytidine treatment.

All presented microarray data is MIAME compliant. The raw microarray data has been deposited in a MIAME compliant database. The microarray data has been deposited at GEO (GEO accession number: GSE17598).

### Real-Time Quantitative PCR

Total RNA was isolated using Trizol; RNA was treated with TURBO DNA-free (Ambion Cat# AM1907). Total RNA (1 µg) was used to make cDNA with the iScript cDNA Synthesis kit (BioRad). Rat Midkine real time PCR was performed with the iQ SYBR Green Supermix (BioRad), and midkine rat specific primers (Forward: CCCAAGATGTAACCCACCAG; Reverse: GCTCACTTCCCAGAATCCC). For SYBR green PCR's, 18S-RNA was used as a reference gene [23] (Forward: GGGAGGTAGTGACGAAAAATAACAAT; Reverse: TTGCCCTCCAATGGATCCT).

Rat sox-2 real time PCR was performed with iQ Supermix (biorad) using Roche universal probe **#**119 (cat. no. 04693531001) and rat specific primers(forward: ATTACCCGCAGCAAAATGAC and Reverse: TTTTTGCGTTAATTTGGATGG). For PCR's with the Roche probes 18S-RNA was used as a reference gene (Probe #22 (cat. no. 04686969001 with primers: Forward: GGTGCATGGCCGTTCTTA; Reverse: TCGTTCGTTATCGGAATTAACC).

The Pfaffl method was used to calculate the normalized gene expression [24]. For each real time PCR analysis the individual sample being examined was used as the test sample in the Pfaffl method. The calibrator sample, for the Pfaffl method, was an equal mixture of cDNA from SRC control cells and 5-Aza-2-deoxycytidine SRC cells. All real time qPCR results are displayed as a ratio of the target gene relative to the reference gene, in a specific test sample, compared to the expression of the target gene relative to the reference gene in the calibrator sample.

### CpG Island Identification

CpG islands in were located by searching the midkine and sox-2 genes in BLAT [25]. Each CpG island was more closely examined using “CpG Island Searcher” [26] and each island was classified as either a high-CpG promoter, an intermediate CpG promoter, or as a low-CpG promoter as previously described [27].

### Analysis of DNA Methylation by Sequencing of Sodium Bisulfite-Treated DNA

Genomic DNA was obtained by digestion with proteinase K (Quiagen) followed by phenol/chloroform extraction, and was subjected to sodium bisulfite treatment to modify unmethylated cytosine to uracil using the ‘CpGenome^™^ DNA Modification Kit’ (Chemicon International, CA). Bissulfite-treated DNA was amplified by a nested-PCR protocol using the primers described in Table S2. PCR was performed in a volume of 25 µl containing PCR Buffer (Qiagen); 1.5 mM of MgCl2 (Qiagen); 200 µM of dNTPs (Invitrogen); 0.32 µM of each primer and 1 U of Hot Start Taq Plus DNA Polymerase (Qiagen). The PCR conditions were: 94°C for 10 min, 94°C for 3 min, 48°C for 3 min, 72°C for 2 min, one cycle; 94°C for 3 min, 50°C for 3 min, 72°C for 2 min, five cycles; and 94°C for 1 min, 52°C for 1 min, 72°C for 1 min, 35 cycles for the first reaction and the same annealing temperatures (48°, 50° and 52°C) for the nested reaction. Amplified products were purified using the Gel Purification Kit (Qiagen) and were ligated to a vector using the TOPO TA Cloning Kit (Invitrogen). Twenty-four positive clones were sequenced for each sample using the vector's forward and reverse primers. DNA sequencing reactions were performed using the ‘DNA dRhodamine Terminator Cycle Sequencing Ready reaction’ kit (Applied Biosystems) and an ABI3730xl sequencer (Applied Biosystems) according to the manufacturer's instructions.

### Invasion Assay

A Membrane Invasion Culture System (MICS) was used to measure the *in vitro* invasiveness of all SRC cell lines as previously described [28]. Briefly, a polycarbonate membrane with 10-um pores was uniformly coated with a defined matrix. Both upper and lower wells of the chamber were filled with RPMI. SRC cells were seeded into upper wells at a concentration of 5×10^5^ cells per well. After a 24-hour incubation in a humidified incubator at 37°C with 5% CO_2_, cells that had invaded through the basement membrane were collected, stained, and counted by light microscopy [29].

### Statistical Analysis

Analysis of Variance (ANOVA) or two-sample t-test was used to analyze changes of DNA methylation level among different treatment conditions for Line1-S1, Line1-S2, Satellites 1 and 2, respectively. Tukey's method or Dunnett method was used to adjust p-values due to multiple comparisons in ANOVA analysis.

A linear regression method was applied to analyze tumor weight between two tumor groups (SRC Control and SRC 5AZA) after adjusting for the number of cells injected. Tumor weight and number of cells were transformed using the logarithm so that data distribution was appropriate for the analysis methods used.

We used 0.05 as the significance level for comparisons. SAS 9.1 and R software was used for data analysis and graphing.

## Results

### 5-Aza-2-Deoxycytidine Induces Hypomethylation of LINE1 and Satellites 1 and 2

Methylation levels of cytosines in CpG dinucleotides of repetitive elements has been used as a surrogate marker for genome-wide methylation [30],in this study, LINE (Long Interspersed Element) 1 and Satellites 1 and 2 were selected as surrogate methylation markers. Rat specific pyrosequencing assays were designed to examine methylation levels of these repetitive sequences throughout the genome. The pyrosequencing assays were used to determine the methylation levels in untreated cells, in cells treated with a low dose of 5-Aza-2-deoxycytidine, and in cells that were treated with 5-Aza-2-deoxycytidine followed by an additional recovery period without the drug (30 days).

**Figure 1:**
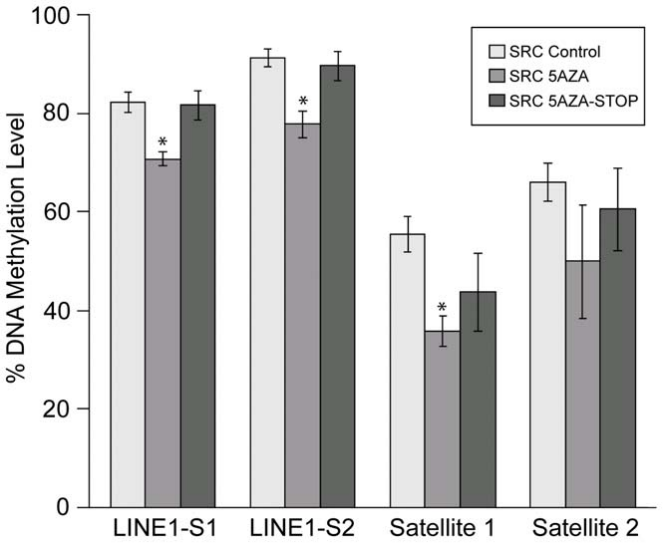
Pyrosequencing of LINE and Satellite 1 and 2 *in vitro*. For each experiment the methylation pattern was analyzed in SRC untreated control cells (SRC-Control), SRC cells treated with 5-Aza-2-deoxycytidine for 5 passages (SRC 5AZA), and in SRC cells that were treated with 5-Aza-2-deoxycytidine for 5 passages and then allowed to grow for 5 additional passages without treatment (SRC 5AZA-STOP). Pyrosequencing assay: LINE1-S1; [2481 CpG's], LINE1-S2; [3308 CpG's]; Satellite I [15CpG's], and Satellite II [411 CpG's]. Treatment of MSCV3-LTC chondrosarcoma cells with 5-Aza-2-deoxycytidine leads to demethylation that can be detected throughout the genome. Altered DNA methylation patterns can be detected several weeks following removal of 5-Aza-2-deoxycytidine treatment. Bars represent the average DNA methylation % of three biologic replicates, and error bars represent the standard deviation of these replicates. ‘*’ Indicates values that are significantly different than the “SRC Control” sample (p<.05).

The level of DNA methylation in LINE, Satellite 1 and Satellite 2 regions of SRC cells decreases following 5-Aza-2-deoxycytidine treatment (Figure 1). The SRC cells were grown for 30 additional days after removal of 5-Aza-2-deoxycytidine. Following withdrawal of the drug, methylation was restored to levels that were indistinguishable from those of control cells based on the LINE1 and Satellite 2 assays (Figure 1). Methylation was partially restored for Satellite 1, but it did not completely regain the level that was observed in control cells. This result suggests that the demethylating effects of 5-Aza-2-deoxycytidine treatment may persist after five additional passages (30 days) without the drug.

### Invasion Assay

**Figure 2:**
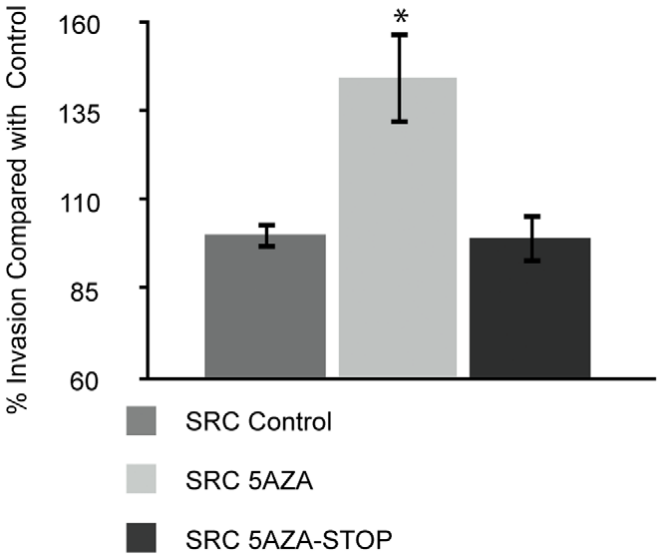
5-Aza-2-deoxycytidine treatment increases the invasiveness of rat chondrosarcoma cells. Invasiveness was measured in control SRC cells (SRC Control), SRC cells that were treated for 5 passages with 5-Aza-2-deoxycytidine (SRC 5AZA), and SRC cells 5-with 5 passages Aza-2-deoxycytidine and then grown for 5 additional passages without treatment (5AZA-STOP). The invasiveness was calculated for all samples and the results are displayed as experimental sample compared to the untreated control SRC cells (100% invasion). The bar represents the average invasion indices of three biologic replicates, and the error bars represent the standard deviation of the biologic replicates. ‘*’ Indicates values that are significantly different than the “SRC Control” sample (p<.05).

The invasiveness of the SRC cells increased 40% following 5-Aza-2-deoxycytidine treatment (Figure 2). Thirty days post-removal of treatment, the invasive activity dropped to a level that was indistinguishable from that of control cells. The invasion assays demonstrated that 5-Aza-2-deoxycytidine-induced DNA hypomethylation leads to an increase in the *in vitro* invasiveness of SRC cells, and that following withdrawal of the drug, the invasive activity of the SRC cells returns to the levels observed for control cells.

### Microarray Analysis

**Figure 3:**
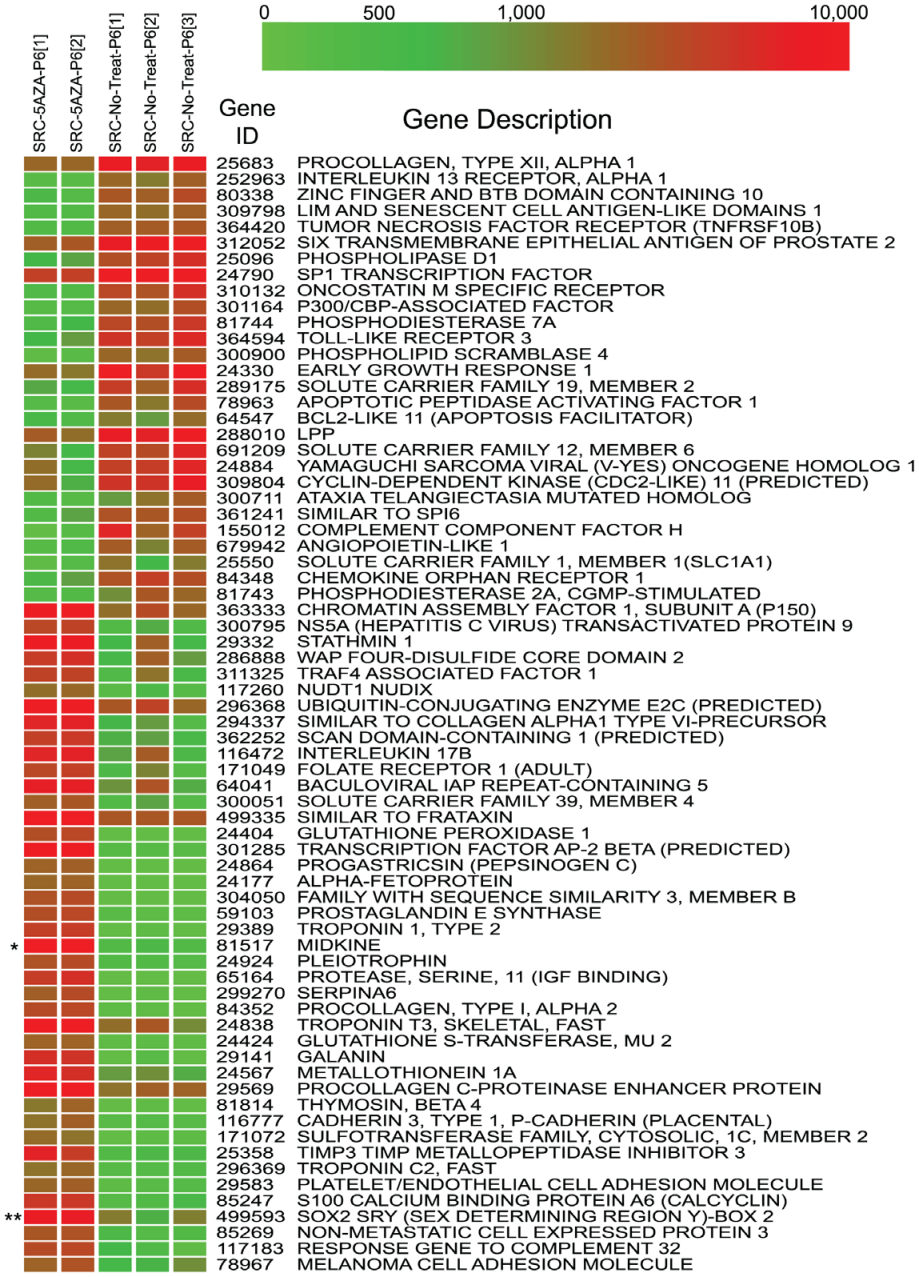
Heat map of differentially expressed genes between SRC cells treated 5-Aza-2-deoxycytidine and untreated control SRC cells. Genes with at least a 5-fold difference were selected for analysis using the pathway program Ingenuity. Ingenuity revealed that, of the 977 differentially expressed genes (603 genes upregulated and 374 downregulated), 135 were identified as cancer related. A subset of these cancer related genes (see Materials and Methods; see Table S3 for complete gene list and expression values) was then used for hierarchical clustering, and the results of that clustering are presented in this figure. Each vertical column represents microarray hybridizations from separate individual experiments. Microarray hybridizations were carried out on SRC cells treated with 5-Aza-2-deoxycytidine for 5 passages (SRC-5-AZA-P6 [1] and [2]), and microarray hybridizations were also carried out on SRC cells grown for 5 passages without 5-Aza-2-deoxycytidine treatment (SRC-No-Treat-P6 [1], [2], and [3]). ‘*’ Indicates midkine and ‘**’ indicates sox-2 in the heat map. The color bar corresponds the to the expression level in relative fluorescent units.

Based on the invasion assays it was hypothesized that the 5-Aza-2-deoxycytidine may alter gene expression of the SRC cells. Microarray analysis was carried out to identify changes in gene expression in untreated and in treated cells. The expression level of several genes increased after treatment with 5-Aza-2-deoxycytidine (Figure 3). Data analysis revealed that 977 genes (603 genes upregulated and 374 downregulated) exhibited a 5-fold expression difference in the untreated and treated SRC cells (see Table S3 for gene list). The pathway-mapping program, Ingenuity, was used to analyze the group of genes with a 5-fold difference in expression (See Table S4 for separate pathway analysis of up- and down regulated genes). Ingenuity revealed that the differentially expressed genes might play a role in several cancer-relevant pathways (Table 1). The top pathway, Cancer (135 genes), was selected for further analysis. A subset of the cancer related genes with a 5-fold difference are shown in a heat map (Figure 3). As illustrated by the heat map, 5-Aza-2-deoxycytidine treatment can lead to the alterations in the expression of genes that may play a role in different aspects of cancer, ranging from cell growth and proliferation, to cell cycle control, and to cell death.

**Table 1:** Top pathways altered following 5-Aza-2-deoxycytidine treatment.

Rank	Function and Diseases	p-value	#Molecules
1	Cancer	9.84E-06	135
2	Cellular Growth and Proliferation	9.84E-06	68
3	Gastrointestinal Disease	9.84E-06	14
4	Nervous System Development and Function	2.23E-05	28
5	Ophthalmic Disease	1.21E-04	11
6	Cellular Function and Maintenance	3.74E-04	17
7	Reproductive System Development and Function	7.29E-04	11
8	Reproductive System Disease	7.40E-04	55
9	Cell Cycle	9.11E-04	20
10	Cell Death	1.16E-03	34

The list of genes with a 5-fold difference (977 genes) in gene expression between untreated SRC control cells and 5-Aza-2-deoxycytidine treated cells was analyzed using Ingenuity Pathway Analysis software. The top 10 functions and diseases are shown in the table. The function, its associated p-value, and the number of molecules in the specific pathway are shown.

**Figure 4:**
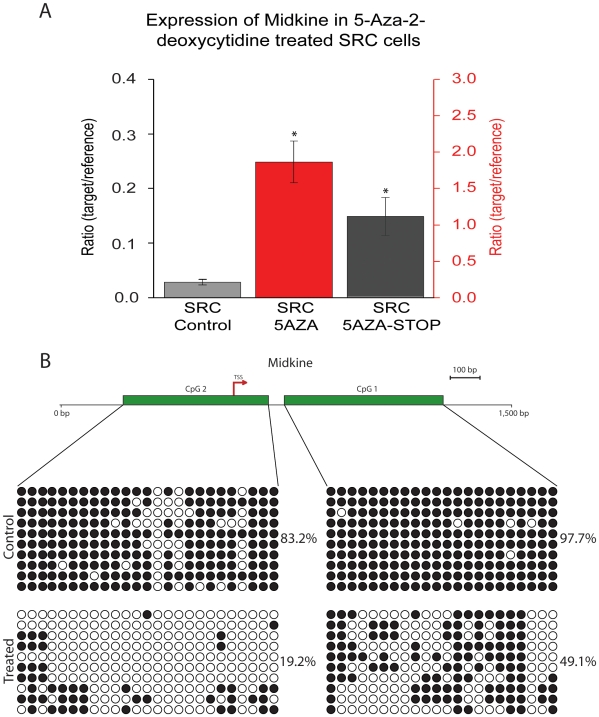
Expression and epigenetic analysis of midkine. (A) Quantitative real time PCR analysis of midkine expression in control SRC cells (SRC Control), SRC cells that were treated for 5 passages with 5-Aza-2-deoxycytidine (SRC 5AZA), and SRC cells 5-with 5 passages Aza-2-deoxycytidine and then grown for 5 additional passages without treatment (5AZA-STOP). Treatment with 5-Aza-2-deoxycytidine induces midkine expression. Five passages following 5-Aza-2-deoxycytidine removal the expression of midkine has dropped but it is greater than that of untreated control cells. Bars represent the average expression of three biologic replicates, and error bars represent the standard deviation of these replicates. ‘*’ Indicates values that are significantly different than the “SRC Control” sample (p<.05). Note that for graphical representation two different vertical scale bars are shown; the vertical scale bar on the left corresponds to the SRC Control and SRC 5AZA-STOP samples, and the vertical Scale bar on the right corresponds with the SRC 5AZA-STOP sample. (B) Schematic representation of analyzed CpG islands in relation to the midkine transcriptional start site (TSS). Green bars indicate regions that were targeted for bisulfite sequencing. Bisulfite sequencing of midkine CpG Island 1 and CpG Island 2. Each row indicates an individual cloned sequence. Circles represent CpG sites. Black circles indicate a methylated CpG site and white circles indicate a unmethylated CpG site. These changes in methylation of the midkine promoter were confirmed by pyrosequencing (Figure S3A). These results demonstrate that 5-Aza-2-deoxycytidine treatment leads to the hypomethylation of CpG islands that span regions of the rat midkine gene.

**Figure 5:**
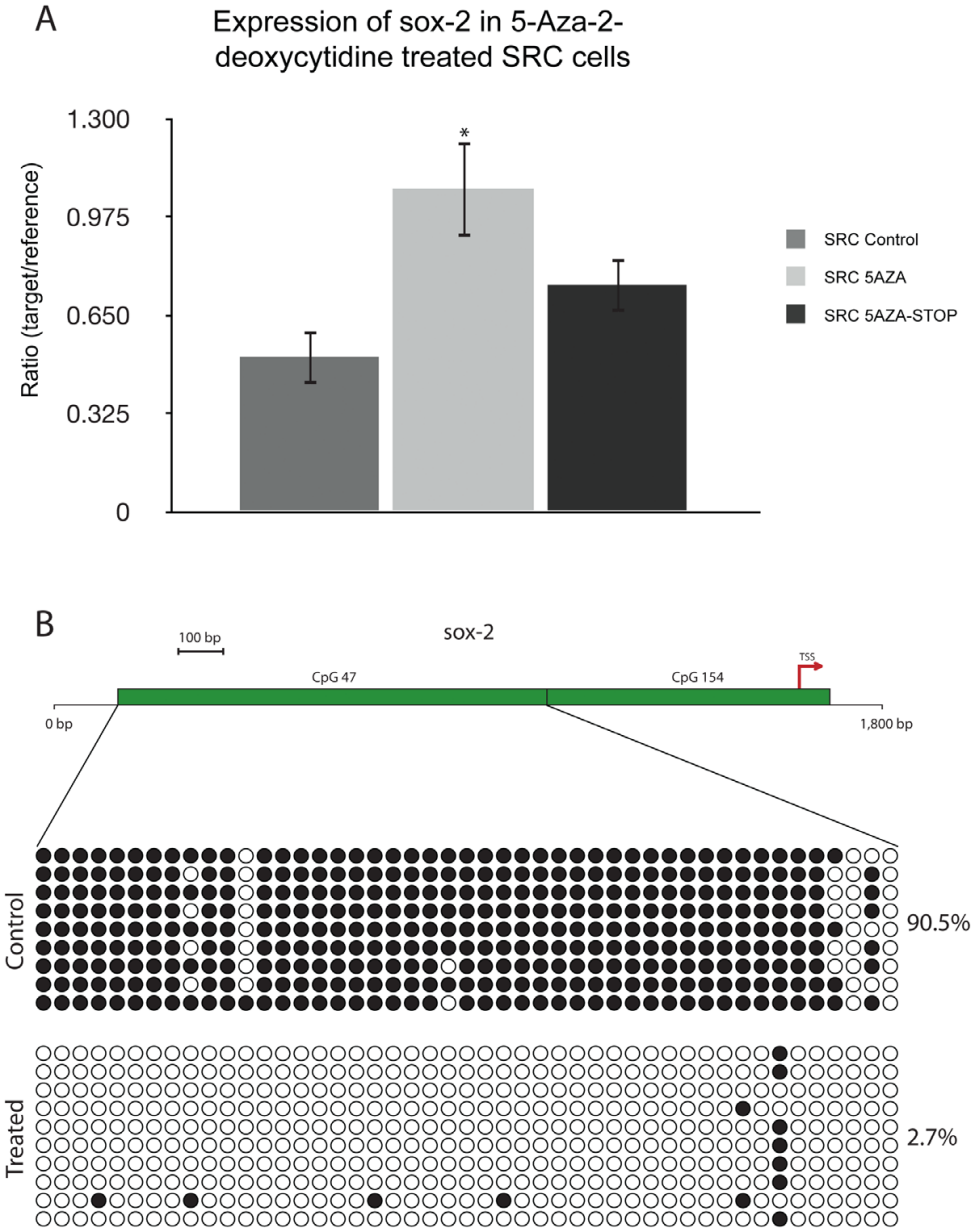
Expression and epigenetic analysis of sox-2. (A) Quantitative real time PCR analysis of sox-2 expression in control SRC cells (SRC Control), SRC cells that were treated for 5 passages with 5-Aza-2-deoxycytidine (SRC 5AZA), and SRC cells 5-with 5 passages Aza-2-deoxycytidine and then grown for 5 additional passages without treatment (5AZA-STOP). Treatment with 5-Aza-2-deoxycytidine induces sox-2 expression. Five passages following 5-Aza-2-deoxycytidine removal the expression of sox-2 has dropped. Bars represent the average expression of three biologic replicates, and error bars represent the standard deviation of these replicates. ‘*’ Indicates values that are significantly different than the “SRC Control” sample (p<.05). (B) Schematic representation of analyzed CpG islands in relation to the sox-2 transcriptional start site (TSS). Green bars indicate regions that were targeted for bisulfite sequencing. Bisulfite sequencing of sox-2 CpG Island 47 and CpG Island 154. Each row indicates an individual cloned sequence. Circles represent CpG sites. Black circles indicate a methylated CpG site and white circles indicate an unmethylated CpG site. CpG 47 Island was methylated in untreated SRC cells but following 5-Aza-2-deoxycytidine treatment it became hypomethylated. These changes in methylation of the sox-2 promter were confirmed by pyrosequencing (Figure S3B). CpG Island 154 was not methylated in either control or treated cells (Figure S4).

One potential explanation of the microarray results is that 5-Aza-2-deoxycytidine treatment results in the derepression of genes that were epigenetically silenced. As a result, we may observe an increase or a decrease in expression (e.g. derepression of a negative regulator). To examine this possibility, expression and methylation analyses were performed on two of the cancer related genes (Figures 4 and 5). The genes, midkine and sox-2, were selected on the basis of their differential expression compared to control cells, and because they have CpG islands in their promoter regions (we have previously demonstrated that both sox-2 and midkine are not expressed in the control tissue, normal rat articular cartilage, data not shown; GEO: GSM25926). Midkine and sox-2 were also selected because they are developmentally regulated genes, and studies have indicated that these genes may play functional roles in stem cells [31], [32]. Therefore, we wanted to determine if 5-Aza-2-deoxycytidine-induced DNA hypomethylation could lead to the expression of stem cell related genes in the SRC cells.

### Midkine and Sox-2

The increase in expression of midkine (Figure 4A) and sox-2 (Figure 5A), following exposure to 5-Aza-2-deoxycytidine, was confirmed by real-time quantitative RT-PCR. The expression of each midkine and sox-2 decreased to a level that is slightly higher than that of control cells thirty days following discontinuation of 5-Aza-2-deoxycytidine treatment. These data suggest that midkine and sox-2 expression increases as a result of exposure to 5-Aza-2-deoxycytidine, and that 30 days after discontinuation of 5-Aza-2-deoxycytidine *in vitro*, the expression of these genes begins to decrease. Although their expression level decreases, both midkine and sox-2 are expressed at levels that are higher than those observed in untreated control cells (Figures 4A and 5A). This suggests that SRC cells may continue to express midkine and sox-2 at high levels for at least 30 days following removal of 5-Aza-2-deoxycytidine.

Both midkine and sox-2 contain CpG islands at their transcription start sites. The CpG islands in the promoters of midkine and sox-2 can be classified as intermediate CpG islands, which is relevant because the activity of promoters containing intermediate CpG islands correlates negatively with their methylation status [27]. Additionally, intermediate CpG islands may be preferential targets for *de novo* methylation in somatic cells during development [27].

The methylation statuses of both midkine and sox-2 CpG islands were examined using bisulfite sequencing (Figures 4B and 5B). Two CpG islands were identified in the rat midkine gene. One CpG island encompasses the midkine transcriptional start site and the other is slightly downstream from it (Figure 4A). Both midkine CpG islands were heavily methylated in untreated SRC cells, and they became hypomethylated in 5-Aza-2-deoxycytidine treated cells (Figure 4B).

Two CpG islands were also examined for sox-2 (Figure 5A). The CpG island encompassing the sox-2 transcriptional start site (CpG 154) was not methylated in either control or treated cells (Figure S4). However, a CpG island (CpG 47) located less than 1 kb upstream of the sox-2 transcriptional start site was methylated in untreated SRC cells. Following 5-Aza-2-deoxycytidine, this CpG island became hypomethylated (Figure 5B).

These results suggest that 5-Aza-2-deoxycytidine treatment can lead to the demethylation of CpG islands at or near the transcriptional start site of midkine (Figures 4B) and sox-2 genes (Figure 5B). The decrease in CpG island methylation was accompanied by an increase in the expression of sox-2 and midkine (Figures 4A and 5A), consistent with the hypothesis that methylation may play a role in the regulation of these genes.

### *In Vivo* Tumor Formation

**Figure 6:**
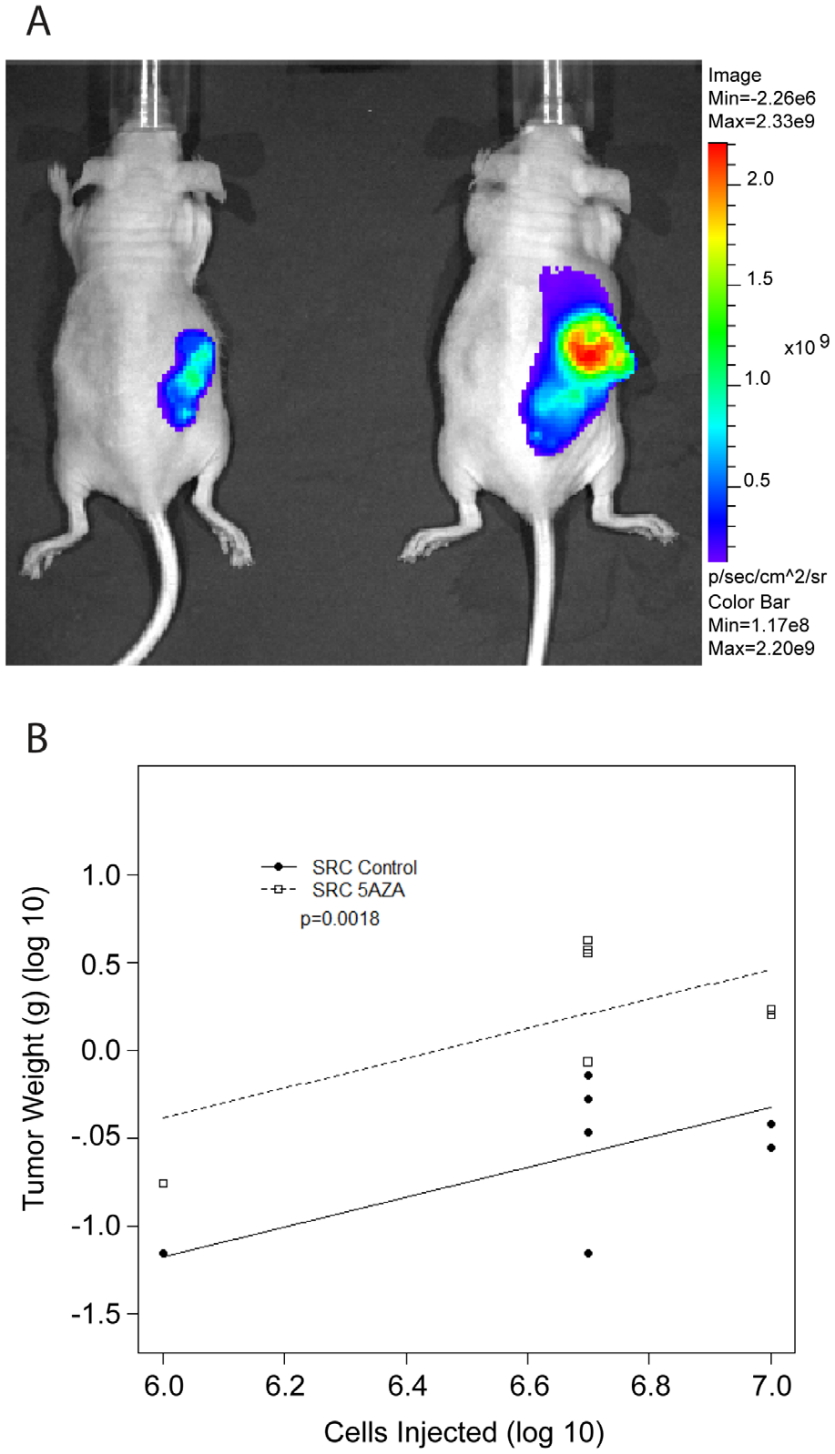
5-Aza-2-deoxycytidine treated SRC cells produced larger tumors than untreated SRC cells. (A) *In vivo* bioluminescent imaging of SRC cells in nude mice. 5×10^6^ Control cells [animal a; left] and 5×10^6^ 5-Aza-2-deoxycytidine treated cells [animal B; right] were injected subcutaneously. This image was collected 6 weeks after tumor induction. This Image corresponds to animal 3a and 3b in Table S5. (B) Summary of *in vivo* SRC injections. Tumors induced with 5-Aza-2-deoxycytidine-treated SRC cells produced larger tumors than the tumors induced with SRC control cells. A linear regression method was applied to analyze tumor weight between two tumor groups (SRC Control and SRC 5AZA) after adjusting for the number of cells injected. For graphical representation the tumor weights and the number of cells injected was log transformed. p-value is for comparison of the two tumor groups (SRC Control and SRC 5AZA), and indicates that there is a significant difference in tumor weight between the two groups. Results are shown for 7 animals with tumors induced from untreated cells (SRC control) and for 7 animals with tumors induced from 5-Aza-2-deoxycytidine-treated cells (SRC 5AZA). Detailed *in vivo* tumor summary is presented in Table S5.

SRC cells were transplanted into nude mice to test tumorigenicity following 5-Aza-2-deoxycytidine-induced DNA hypomethylation. The SRC cell line used for all aforementioned experiments stably expresses luciferase, which enables tumor growth to be examined *in vivo*. SRC cells treated with 5-Aza-2-deoxycytidine produced larger tumors than those induced with control SRC cells (Figure 6A and 6B).

**Figure 7:**
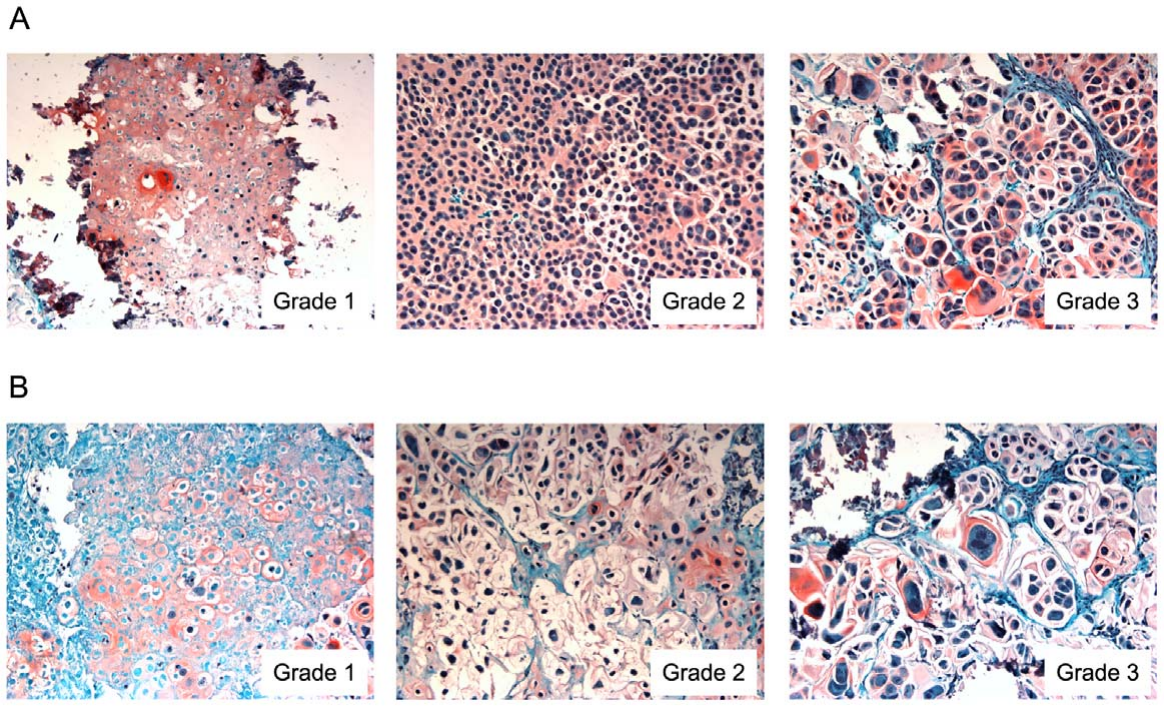
Photomicroscopy of histological sections obtained from SRC tumors (20x magnification). (A) Subcutaneous tumor induced from untreated SRC control cells. (B) Subcutaneous tumor induced from 5-Aza-2-deoxycytidine SRC cells. Approximately 60 days following tumor induction animals were sacrificed and tumors were removed for histology. Tumors from the SRC control cells and the 5-Aza-2-deoxycytidine cells showed considerable heterogeneity. There was no clear histological difference between tumors initiated from control cells or treated cells. Low grade (Grade 1) – Small nuclei with low variation in size and abundant cartilage matrix. Intermediate grade (Grade 2) – Higher cellularity, larger nuclei with increased atypia and hyperchromasia. High grade (Grade 3) – Pleomorphic cells with greater degree atypia and nuclear size. The SRC cells are stained with Safranin O (red).

Subcutaneous tumors were induced with 1×10^6^, 5×10^6^, 7×10^6^, or 10×10^6^ cells. In preparation for injections, SRC cells were treated *in vitro* for 30 days with 5-Aza-2-deoxycytidine, after which the treatment was stopped and the cells were transplanted into nude mice. Control tumors were induced with untreated SRC cells. Tumors were resected sixty days after transplantation. As it has been documented with human chondrosarcoma [33], [34], the SRC cells produced tumors with varying degrees of heterogeneity (Figure 7A and 7B). Albeit relevant, histological grading is not predictive of outcome [35], and no markers of prognostic value have been identified to date for human chondrosarcoma [36]. Hence, we examined another characteristic of the tumor, tumor weight, which demonstrated that 5-Aza-2-deoxycytidine treated cells produced larger tumors than those derived from untreated cells (Figure 6B and Table S5).

Histological analysis detected the presence of SRC cells in the lungs of mice injected with control cells and in the lungs of mice injected with 5-Aza-2-deoxycytidine treated cells (Table S5). However, it should be noted that SRC cells were detected macroscopically in the lungs of mice injected with 5-Aza-2-deoxycytidine-treated cells (3 out of 9 mice; Figure S2). SRC cells could not be detected macroscopically in the lungs of mice injected with untreated control cells. These results indicated that 5-Aza-2-deoxycytidine-treated SRC cells may grow more aggressively than untreated SRC cells in the lung of subcutaneously injected mice.

Taken together, the *in vivo* analyses demonstrated that DNA hypomethylation, induced by 5-Aza-2-deoxycytidine, led to the formation of more aggressive tumors than the tumors formed from untreated control SRC cells both locally, at the site of injection, and distantly, in the lungs.

### Methylation of SRC Cells *In Vivo*

**Figure 8:**
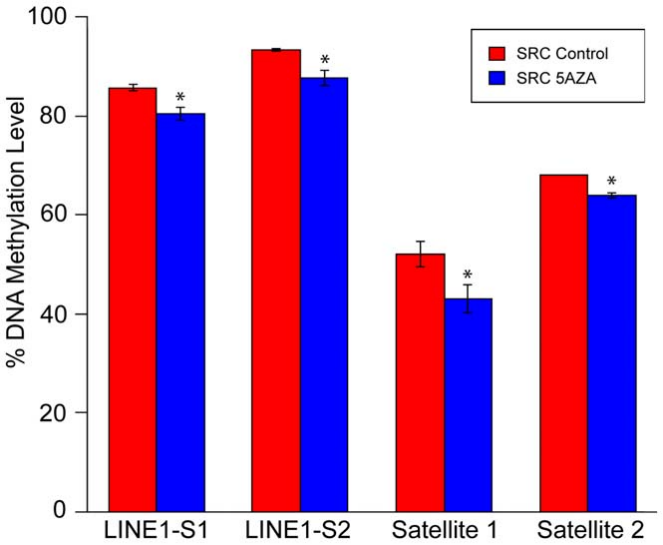
Pyrosequencing of LINE and Satellite 1 and 2 *in vivo*. Pyrosequencing results are also displayed from tumor samples: tumors initiated from untreated SRC cells (SRC Control) and tumors initiated from SRC cells that were treated for 5 passages with 5-Aza-2-deoxycytidine (SRC 5AZA). Results are shown for 3 SRC Control tumors and 3 SRC 5AZA tumors. Pyrosequencing assay: LINE1-S1; [2481 CpG's], LINE1-S2; [3308 CpG's], Satellite I [15CpG's], Satellite II [411 CpG's]. In all regions examined by pyrosequencing the SRC 5AZA tumors have a significantly lower level of methylation than the SRC Control tumors. Bars represent the average DNA methylation % of three biologic replicates, and error bars represent the standard deviation of these replicates. ‘*’ Indicates values that are significantly different than the “SRC Control” sample (p<.05).

The tumors derived from untreated SRC cells were more methylated than the tumors derived from 5-Aza-2-deoxycytidine treated cells (Figures 8). *In vivo* the 5-Aza-2-deoxycytidine treated cells were injected and allowed to grow *in vivo* without treatment for 60 days. After 60 days of growth *in vivo*, tumors derived from 5-Aza-2-deoxycytidine-treated SRC cells exhibited a significantly lower level of methylation (Figure 8; LINE-1, LINE1-S2, Satellite 1, and Satellite 2) than that of the tumors derived from untreated control cells. This result is of note since *in vitro* the SRC cells were treated for 30 days and subsequently grown *in vitro* for 30 days without treatment, but these cells did reestablish methylation levels that were similar to control cells (Figure 1; LINE-1, LINE1-S2, and Satellite2). These results suggest that 5-Aza-2-deoxycytidine treated SRC cells more efficiently reestablish hypermethylation *in vitro* than *in vivo*.

## Discussion

Aberrant DNA hypermethylation has been observed in a variety of cancers including chondrosarcoma [37], [38], [39]. 5-Aza-2-deoxycytidine treatment is thought to lead to the reactivation of aberrantly hypermethylated genes [40], and treatment of leukemias with 5-Aza-2-deoxycytidine has been shown to have clinical benefits [7]. However, genome-wide hypometylation has also been observed in several types of cancer[8], and it has been suggested that DNA hypomethylation may play a role in tumorigenesis [10], [11]. Although 5-Aza-2-deoxycytidine does have clinical benefits, one potential concern with a drug that induces DNA hypomethylation is the possibility that, in addition to reintroducing the expression of abnormally silenced genes, it may also lead to the expression of genes that are normally epigenetically silenced or it may lead to an increase of illegitimate transcription events [18]. Genome-wide derepression of transcription of genes that are normally epigenetically silenced is likely to have a dramatic impact in cancer cells that already possess abnormal genetic, epigenetic, or gene expression profiles**.**

In this study, we examined the effect of 5-Aza-2-deoxycytidine-induced genome-wide hypomethylation on SRC cells *in vitro* and *in vivo*, using pyrosequencing assays. As expected, treatment with 5-Aza-2-deoxycytidine led to a decrease in the global methylation levels of SRC cells. This decrease in methylation was accompanied by an increase in the invasiveness of the SRC cells *in vitro*. Subsequent global gene expression analysis revealed that 5-Aza-2-deoxycytidine treatment leads to (abnormal) expression of several cancer related genes.

More detailed analysis of two of the cancer related genes, sox-2 and midkine, confirmed that their expression levels increased following 5-Aza-2-deoxycytidine treatment. Methylation analysis of CpG islands at their transcription start site revealed that these genes were methylated in control cells and that they lost methylation after treatment with 5-Aza-2-deoxycytidine. This result suggests that loss of methylation may play a role in the activation of both sox-2 and midkine in 5-Aza-2-deoxycytidine-treated SRC cells. It is noteworthy that sox-2 and midkine contain “intermediate-type” CpG islands [27]. Transcriptional activity of genes with such type of CpG islands is known to correlate negatively with their level of methylation[27]. The expression of these genes may, at least in part, explain the increase in invasiveness following 5-Aza-2-deoxycytidine treatment, as both sox-2 and midkine may play roles in tumor progression [41], [42], [43].

Sox-2 and midkine may also have a function in stem cells. Midkine is involved in the growth of neuronal stem cells [32], and the expression of sox-2 has been shown to be an important factor for restoring somatic cells to a pluripotent state [31]. An intriguing possibility is that 5-Aza-2-deoxycytidine may induce the expression of genes or networks that allow the cells to acquire stem cell-like properties. Pathway analysis of the differentially expressed genes led to the identification of a network of stem cell related genes that became upregulated following 5-Aza-2-deoxycytidine treatment (Figure S5). Based on the network, sox-2 plays a role in the regulation of Dppa5 [44], Alpha crystallin B [45] and P-cadherin [46], and this is consistent with the microarray data (see Figure 3 and Table S3). Although products of these genes may have functions in stem cells, their role in the SRC cells is not known. It is important to note that these genes may have additional cellular functions. For example, besides their putative roles in stem cells, midkine and Alpha crystallin B may also play a role in drug resistance [47], [48]. These examples demonstrate the complex nature of gene expression changes that occur following 5-Aza-2-deoxycytidine treatment.

A number of genes are also downregulated following 5-Aza-2-deoxycytidine treatment. Among these are genes with diverse cellular functions (see Figure 3 and Table S3). One potential explanation for this observation is that 5-Aza-2-deoxycytidine treatment may lead to the activation of genes that negatively regulate other genes. These negative regulators may include protein-coding genes, as well as microRNA genes that could negatively regulate gene expression [49]. Another intriguing possibility is that 5-Aza-2-deoxycytidine treatment might lead to the expression of other noncoding antisense RNAs that could in turn negatively regulate gene expression [50], [51], [52], [53].

5-Aza-2-deoxycytidine-induced changes in gene expression had a significant impact on the phenotype of the SRC cells as tumors derived from 5-Aza-2-deoxycytidine treated cells produced larger tumors than tumors derived from untreated cells (Figure 6A and 6B). Tumors derived from 5-Aza-2-deoxycytidine treated cells had a lower level of methylation (60 days after tumor induction) than those derived from control untreated cells (Figure 8 This is a notable observation because *in vitro*, 30 days following 5-Aza-2-deoxycytidine removal, the SRC cells had reestablished a methylation level that was similar to that of control cells (Figure 1). It is possible that the *in vivo* microenvironment may provide more favorable growth conditions that would allow selection and/or propagation of hypomethylated cells. *In vitro* cells do not encounter the same selective pressure as *in vivo* cells do, and this difference in selective pressure may provide an explanation as to why the tumor cells maintain a lower level of methylation.

While it can be speculated that the microenvironment may exert some selective pressure on the SRC cells *in vivo*, the possibility cannot be ruled out that the *in vitro* cells may have a faster doubling time than that of the cells *in vivo*. The faster doubling time would presumably allow the *in vitro* cells to more quickly regain DNA methylation, whereas the cells *in vivo* may have a slower doubling time and therefore would require more time for the cells to reestablish the same methylation level that was observed *in vitro*.

It is important to note that 5-Aza-2-deoxycytidine is not currently used for the treatment of human chondrosarcoma, and the treatment schedule presented in this paper was designed for the treatment of cells *in vitro* and therefore it does not match a standard clinical treatment schedule. Consequently, the results obtained with 5-Aza-2-deoxycytidine may be specific for the SRC cell line and the conditions of the experiment.

Despite the nontraditional use of 5-Aza-2-deoxycytidine, our results suggest that genome-wide DNA hypomethylation, induced by 5-Aza-2-deoxycytidine, may actually promote certain aspects of tumorigenesis in SRC cells. This observation may initially seem counterintuitive based on the use of 5-Aza-2-deoxycytidine as a chemotherapeutic agent, but previous studies have demonstrated that 5-Aza-2-deoxycytidine can be mutagenic [54], and that DNA hypomethylation can promote the formation of tumors [10], [11]. Recent studies have also shown that chromatin modifying agents, including 5-Aza-2-deoxycytidine, are capable of inducing pluripotency associated genes [55] and it is possible to speculate that the activation of pluripotency associated genes may have a substantial impact on tumor cells. However, further studies are needed to attain a greater understanding of the effect that these epigenetic modifying drugs have on tumor cells. Finally, additional studies are needed to investigate the specific mechanisms by which genome-wide loss of methylation may promote tumorigenesis.

## Supporting Information

Table S1 Pyrosequencing primer design and PCR conditions
(0.02 MB XLS)

Table S2 Bisulfite treated DNA primer design
(0.04 MB XLS)

Table S3 5-aza-2-deoxycytidine treatment induces gene expression changes in the SRC cells
(0.19 MB XLS)

Table S4 Top pathways upregulated and downregulated following 5-Aza-2-deoxycytidine treatment. The list of genes with a 5-fold difference (977 genes) in gene expression between untreated SRC control cells and 5-Aza-2-deoxycytidine treated cells were further separated into lists of upregulated (603 genes) or downregulated (374 genes) genes. The lists were analyzed using Ingenuity Pathway Analysis software. The top 10 functions and diseases are shown in the table. The function, its associated p-value, and the number of molecules in the specific pathway are shown.
(0.28 MB TIF)

Table S5 In vivo: Subcutaneous transplantation of SRC tumor cells. SRC-MSCV3-LTC Cells were injected into nude mice. The number of cells injected in a given experiment is listed in the table. Control cells were grown for 5 passages without any drug treatment and the cells were injected subcutaneously. Treated cells were grown for 5 passages in the presence of 5-Aza-2-deoxycytidine, treatment was removed, and the cells were injected subcutaneously. Approximately 60 days after subcutaneous cell injections the animals were sacrificed and the tumors were collected. Rows in the table represent individual animals. For each experiment one animal was injected with control cells (A) and a corresponding animal was injected with 5-Aza-2-deoxycytidine treated cells (B). The units for surface radiance are photons/sec/cm2/sr. For certain animals the lungs were saved for histologic analysis and the detection of tumor cells in the lung is noted in the table.
(0.23 MB TIF)

Figure S1 Percent viability of SRC cells following 72 hour and 144 hour incubation with 5-Aza-2-deoxycytidine. (A) Percent viability of SRC cells 72 hours following treatment with 0, 0.1, 0.3, 1.0, 3.0, 10, 30,100, and 300 uM 5-Aza-2-deoxycytidine. (B) Percent viability of SRC cells following treatment with 0, 0.1, 1.0, 10, and 100 uM 5-Aza-2-deoxycytidine. Viability is displayed as the % of the control sample as previously described [22]. Each point on the graph represents the average viability of three biologic replicates, and the error bars represent the standard deviation of the biologic replicates. “***” indicates a significant difference between a given sample and the control sample (0.0 uM, no treatment).
(0.32 MB TIF)

Figure S2 Macrometastasis detected in the lungs of mice injected with 5-Aza-2-deoxycytidine treated SRC cells. Macrometastases were detected in 3 of 9 animals injected with 5-Aza-2-deoxycytidine treated cells, but no macrometastases were detected in the lungs of mice injected with untreated cells. Metastases of varying size were detected in the in the lungs of the same animal. The SRC tumor cells form nodules of different sizes and resemble hyaline cartilage. Lungs from 3 separate mice are displayed in the figure.
(2.22 MB TIF)

Figure S3 Pyrosequencing of the midkine and sox-2 promoter. CpG sites in the midkine(A) and sox-2 (B) promoter sequence are methylated in untreated SRC cells but following 5-Aza-2-deoxycytidine treatment they become hypomethylated. The promoter regions of midkine and sox-2 were analyzed for DNA methylation status. Pyrosequencing was used to analyze bisulfite treated DNA with primers specific for midkine or sox-2. The bar represents the average DNA methylation of technical replicates, and the error bars represent the standard deviation of the technical replicates. ‘*’ Indicates that the values are significantly different than the “SRC Control” sample (p<.05). Five CpG sites were examined with the midkine promoter analysis. Eight CpG sites were examined in the sox-2 analysis. The pyrosequencing analysis for midkine promoter was carried out using the following primers: MDK-2-c-F1: biotin/GTTAAGGTTTTTTTTGTTTTTAGAAT; MDK-2-c-F1: TAAATAACACAAACACAAAAAATCC; (Sequencing primer) MDK-2-c-S1: ACAAACACAAAAATCCC. The pyrosequencing analysis for sox-2 promoter was carried out using the following primers: Sox_2_CpG47-3-F1: ttgtgttaattagtaggggtaatg; Sox_2_CpG47-3-R1: biotin/CAACTTCCTAACATCCCA; (Sequencing primer) Sox_2_CpG47-3-S1: TGTGTTAATTAGTAGGGGTA.
(0.54 MB TIF)

Figure S4 Epigenetic analysis of sox-2. Bisulfite sequencing of sox-2 CpG Island 154. CpG Island 154 was not methylated in either control or treated cells. Schematic representation of analyzed CpG islands in relation to the sox-2 transcriptional start site (TSS). Green bars indicate regions that were targeted for bisulfite sequencing. Each row indicates an individual cloned sequence. Circles represent CpG sites. Black circles indicate a methylated CpG site and white circles indicate an unmethylated CpG site. See Figure 5B for sox-2 CpG Island 47 methylation analysis.
(0.53 MB TIF)

Figure S5 Identification of Sox-2 regulated genes. (A) Genes with a 5-fold difference between control and 5-Aza-2-deoxycytidine treated cells were imported into the pathway program GeneGO. GenGO identified a network whereby sox-2 putatively activates the expression of Dppa5, Alpha crystallin B, and P-cadherin. (B) Upregulation of Dppa5, CyraB, Cdh3, and Sox2 following 5-aza-2-deoxycytidine treatment. Closer examination of the expression analysis indicated that the genes, displayed in Figure S4A, become upregulated following 5-Aza-2-deoxycytidine treatment. The relative expression ratios of Dppa5, Alpha crystallin B, and P-cadherin are similar to that of sox-2, suggesting that sox-2 may play a role in the regulation of these genes. Expression values extracted from normalized microarray data.
(0.09 MB TIF)
