# Supplementary figures and images for: Global Demethylation of Rat Chondrosarcoma Cells after Treatment with 5-Aza-2′-Deoxycytidine Results in Increased Tumorigenicity
Source: PLoS One. 2009 Dec 17;4(12):e8340. doi: 10.1371/journal.pone.0008340 (PMC2790612; doi:10.1371/journal.pone.0008340)

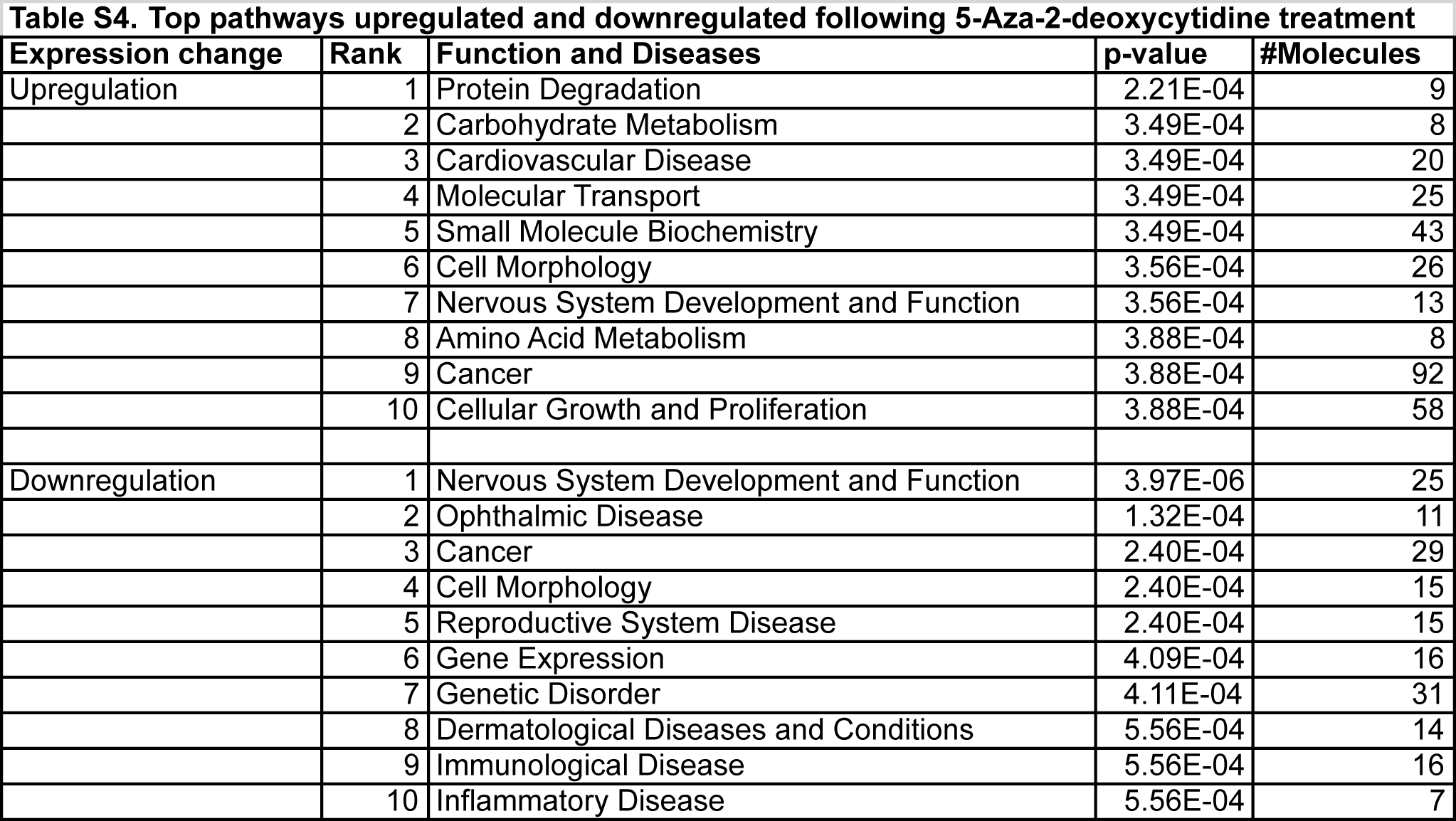

Supplement: Table S4 — Top pathways upregulated and downregulated following 5-Aza-2-deoxycytidine treatment. The list of genes with a 5-fold difference (977 genes) in gene expression between untreated SRC control cells and 5-Aza-2-deoxycytidine treated cells were further separated into lists of upregulated (603 genes) or downregulated (374 genes) genes. The lists were analyzed using Ingenuity Pathway Analysis software. The top 10 functions and diseases are shown in the table. The function, its associated p-value, and the number of molecules in the specific pathway are shown. (0.28 MB TIF) [file pone.0008340.s004.tif]

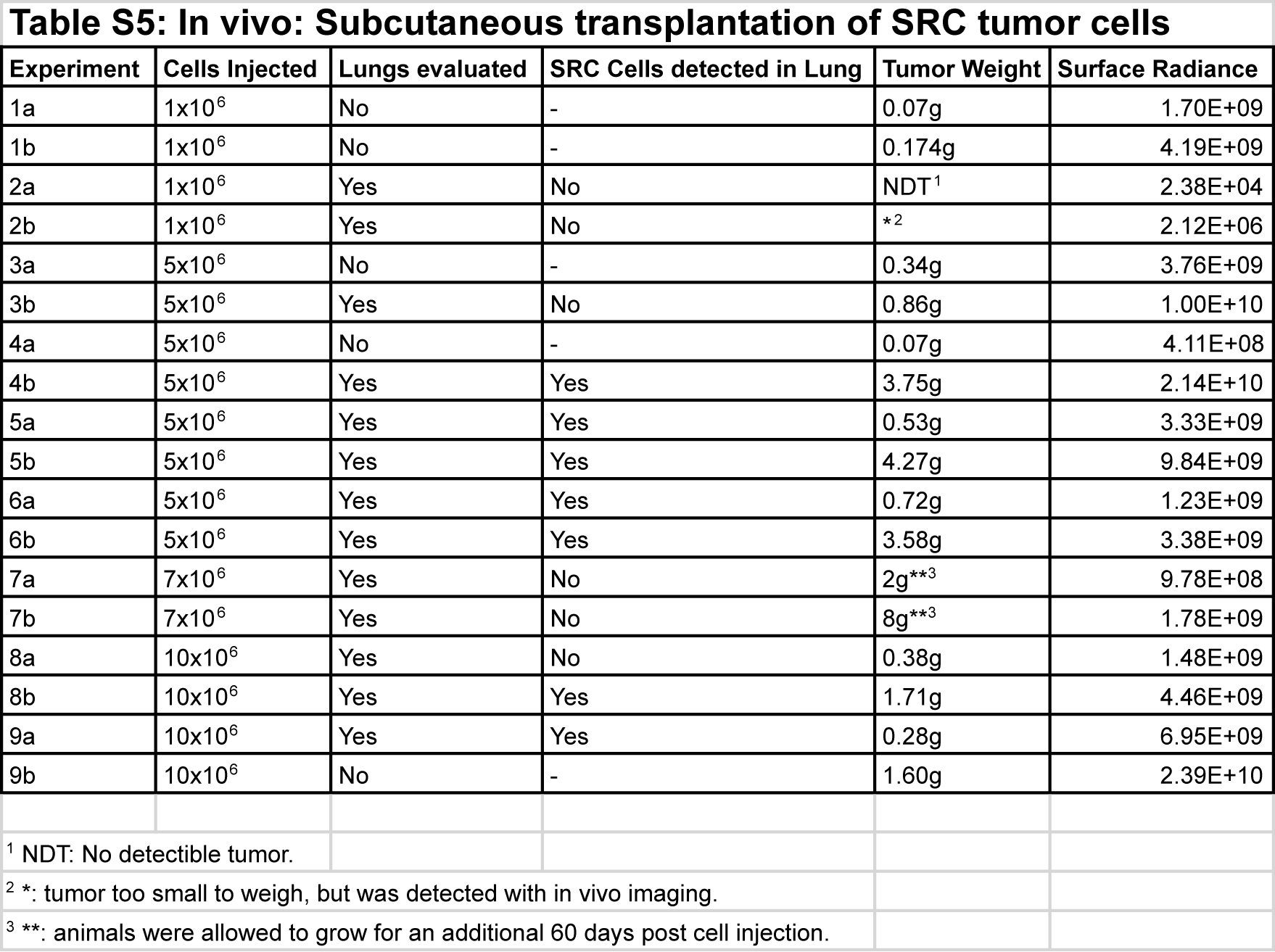

Supplement: Table S5 — In vivo: Subcutaneous transplantation of SRC tumor cells. SRC-MSCV3-LTC Cells were injected into nude mice. The number of cells injected in a given experiment is listed in the table. Control cells were grown for 5 passages without any drug treatment and the cells were injected subcutaneously. Treated cells were grown for 5 passages in the presence of 5-Aza-2-deoxycytidine, treatment was removed, and the cells were injected subcutaneously. Approximately 60 days after subcutaneous cell injections the animals were sacrificed and the tumors were collected. Rows in the table represent individual animals. For each experiment one animal was injected with control cells (A) and a corresponding animal was injected with 5-Aza-2-deoxycytidine treated cells (B). The units for surface radiance are photons/sec/cm2/sr. For certain animals the lungs were saved for histologic analysis and the detection of tumor cells in the lung is noted in the table. (0.23 MB TIF) [file pone.0008340.s005.tif]

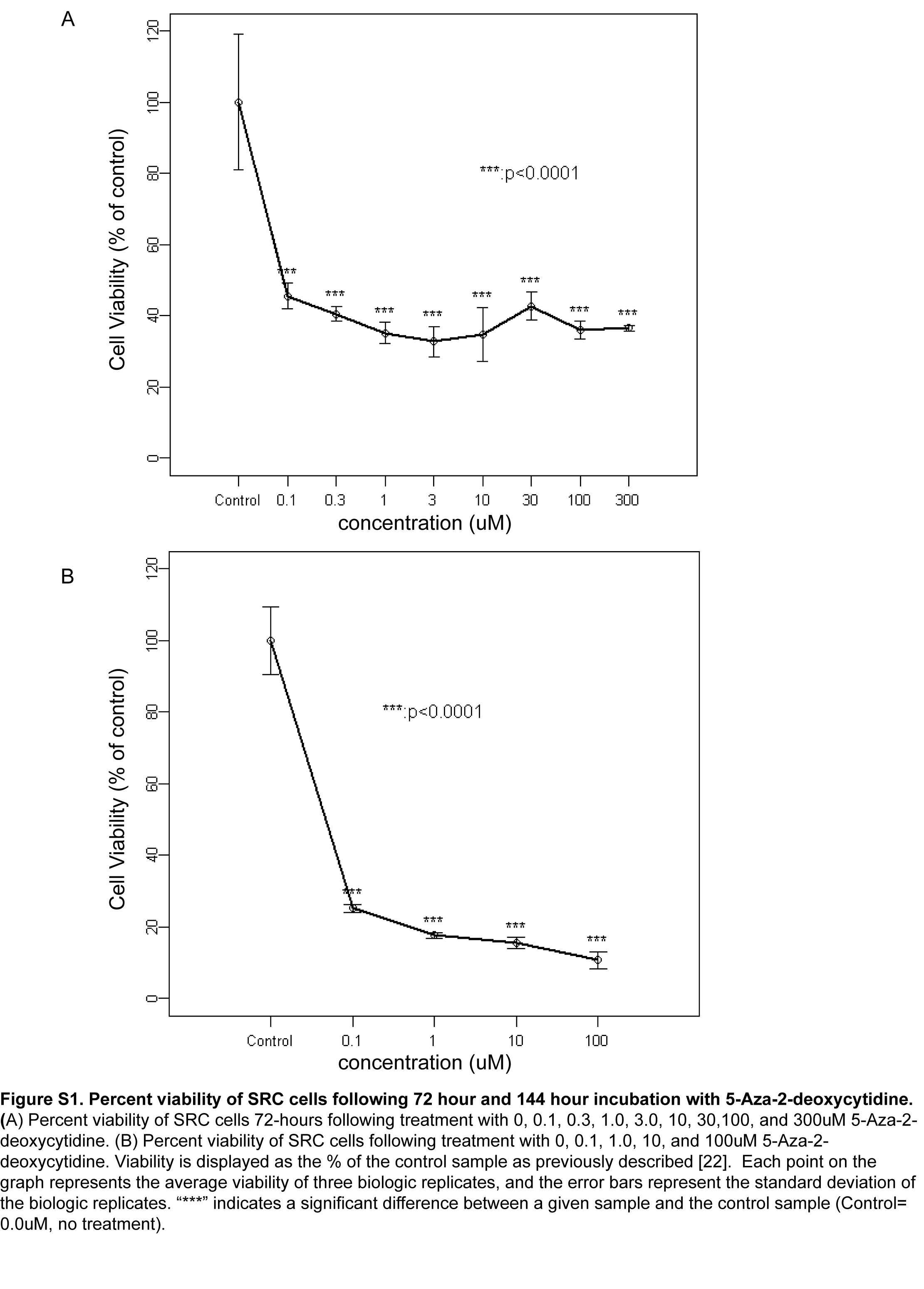

Supplement: Figure S1 — Percent viability of SRC cells following 72 hour and 144 hour incubation with 5-Aza-2-deoxycytidine. (A) Percent viability of SRC cells 72 hours following treatment with 0, 0.1, 0.3, 1.0, 3.0, 10, 30,100, and 300 uM 5-Aza-2-deoxycytidine. (B) Percent viability of SRC cells following treatment with 0, 0.1, 1.0, 10, and 100 uM 5-Aza-2-deoxycytidine. Viability is displayed as the % of the control sample as previously described [22]. Each point on the graph represents the average viability of three biologic replicates, and the error bars represent the standard deviation of the biologic replicates. “***” indicates a significant difference between a given sample and the control sample (0.0 uM, no treatment). (0.32 MB TIF) [file pone.0008340.s006.tif]

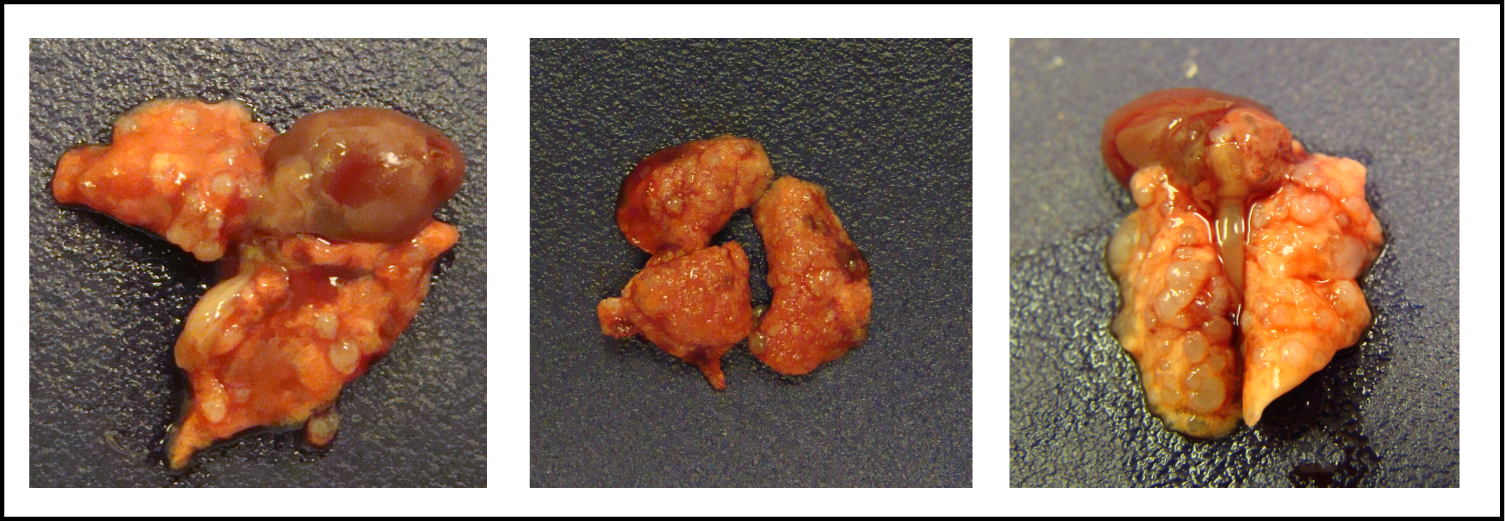

Supplement: Figure S2 — Macrometastasis detected in the lungs of mice injected with 5-Aza-2-deoxycytidine treated SRC cells. Macrometastases were detected in 3 of 9 animals injected with 5-Aza-2-deoxycytidine treated cells, but no macrometastases were detected in the lungs of mice injected with untreated cells. Metastases of varying size were detected in the in the lungs of the same animal. The SRC tumor cells form nodules of different sizes and resemble hyaline cartilage. Lungs from 3 separate mice are displayed in the figure. (2.22 MB TIF) [file pone.0008340.s007.tif]

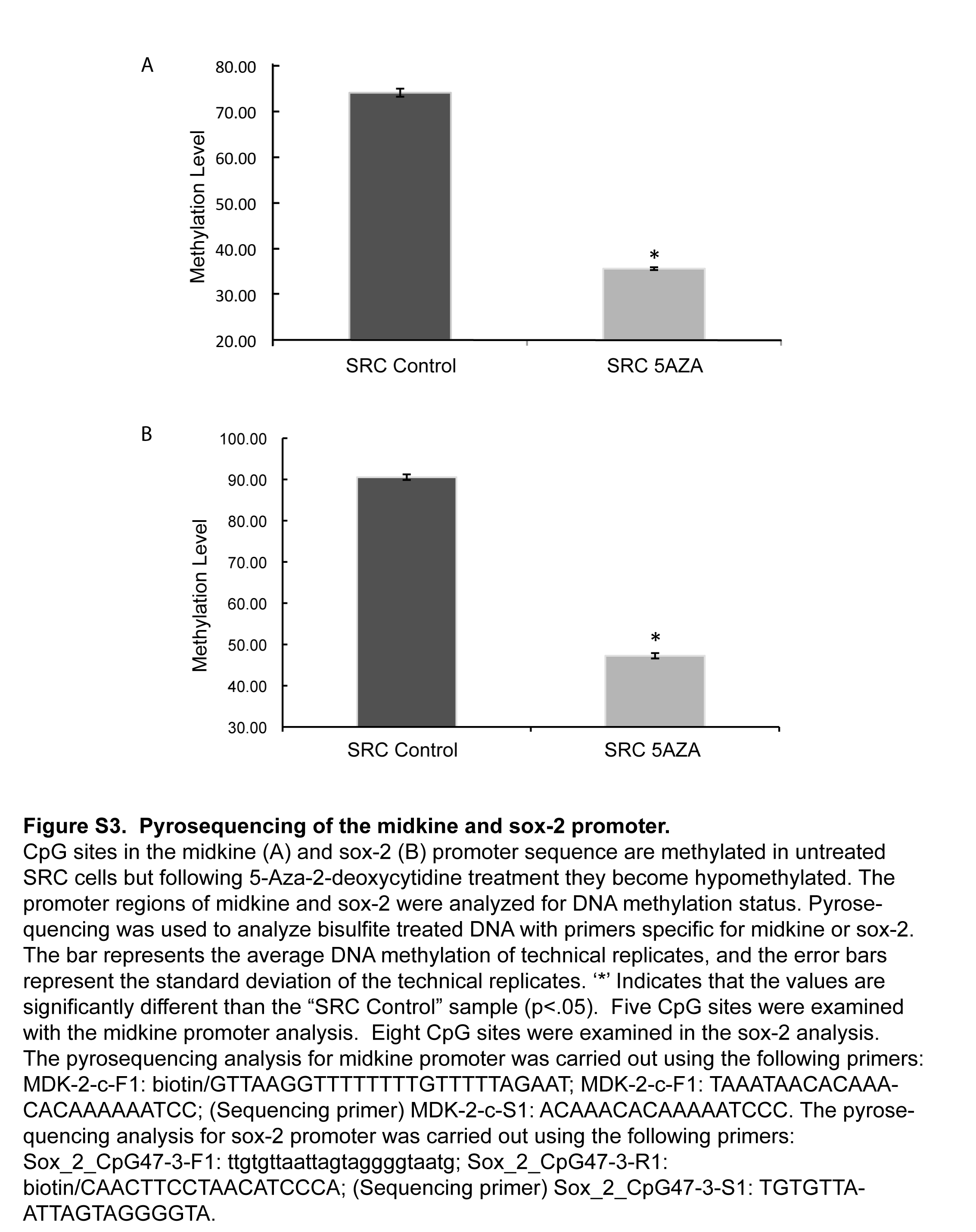

Supplement: Figure S3 — Pyrosequencing of the midkine and sox-2 promoter. CpG sites in the midkine(A) and sox-2 (B) promoter sequence are methylated in untreated SRC cells but following 5-Aza-2-deoxycytidine treatment they become hypomethylated. The promoter regions of midkine and sox-2 were analyzed for DNA methylation status. Pyrosequencing was used to analyze bisulfite treated DNA with primers specific for midkine or sox-2. The bar represents the average DNA methylation of technical replicates, and the error bars represent the standard deviation of the technical replicates. ‘*’ Indicates that the values are significantly different than the “SRC Control” sample (p<.05). Five CpG sites were examined with the midkine promoter analysis. Eight CpG sites were examined in the sox-2 analysis. The pyrosequencing analysis for midkine promoter was carried out using the following primers: MDK-2-c-F1: biotin/GTTAAGGTTTTTTTTGTTTTTAGAAT; MDK-2-c-F1: TAAATAACACAAACACAAAAAATCC; (Sequencing primer) MDK-2-c-S1: ACAAACACAAAAATCCC. The pyrosequencing analysis for sox-2 promoter was carried out using the following primers: Sox_2_CpG47-3-F1: ttgtgttaattagtaggggtaatg; Sox_2_CpG47-3-R1: biotin/CAACTTCCTAACATCCCA; (Sequencing primer) Sox_2_CpG47-3-S1: TGTGTTAATTAGTAGGGGTA. (0.54 MB TIF) [file pone.0008340.s008.tif]

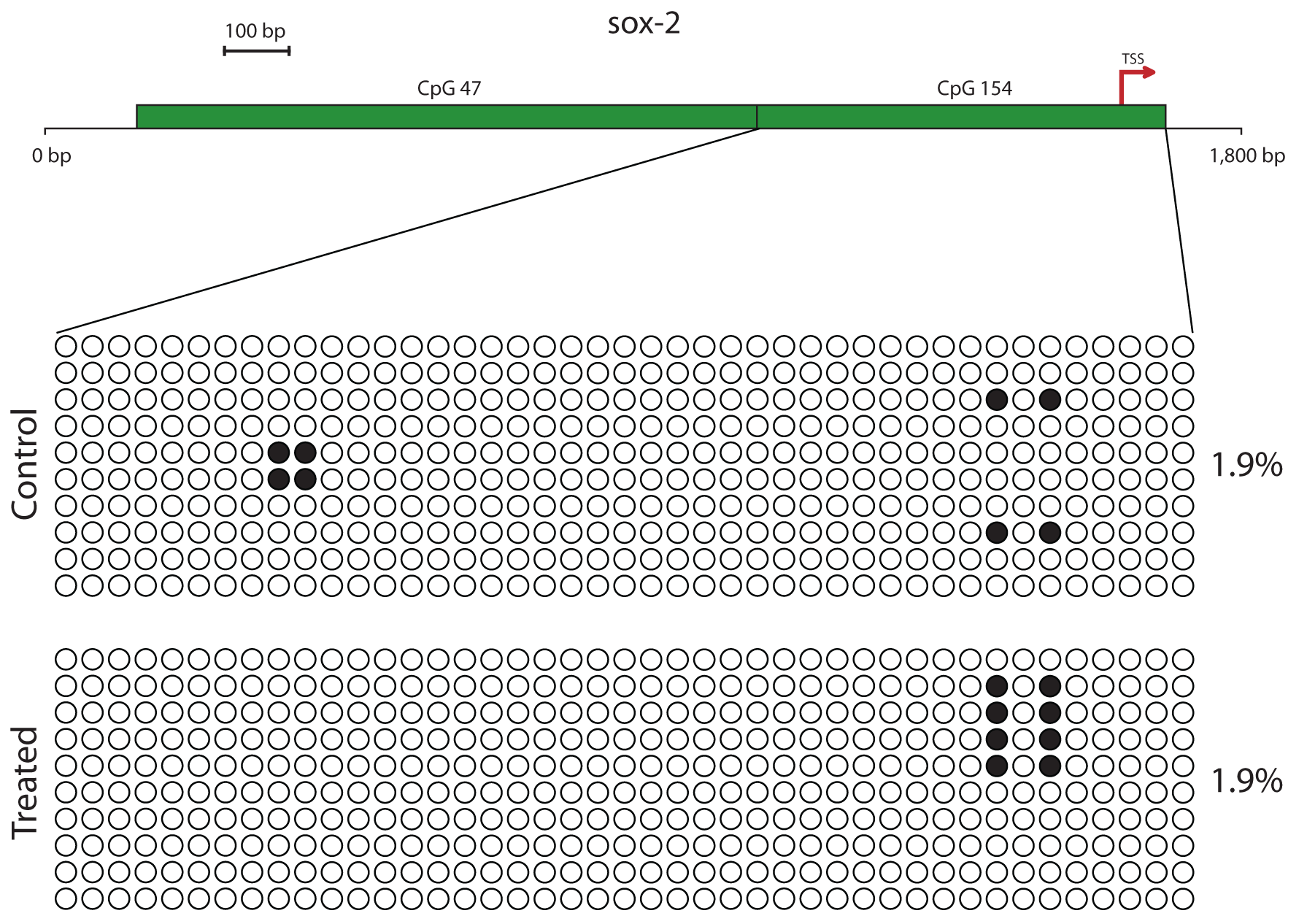

Supplement: Figure S4 — Epigenetic analysis of sox-2. Bisulfite sequencing of sox-2 CpG Island 154. CpG Island 154 was not methylated in either control or treated cells. Schematic representation of analyzed CpG islands in relation to the sox-2 transcriptional start site (TSS). Green bars indicate regions that were targeted for bisulfite sequencing. Each row indicates an individual cloned sequence. Circles represent CpG sites. Black circles indicate a methylated CpG site and white circles indicate an unmethylated CpG site. See Figure 5B for sox-2 CpG Island 47 methylation analysis. (0.53 MB TIF) [file pone.0008340.s009.tif]

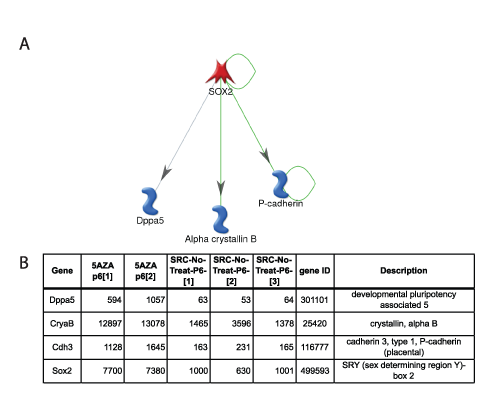

Supplement: Figure S5 — Identification of Sox-2 regulated genes. (A) Genes with a 5-fold difference between control and 5-Aza-2-deoxycytidine treated cells were imported into the pathway program GeneGO. GenGO identified a network whereby sox-2 putatively activates the expression of Dppa5, Alpha crystallin B, and P-cadherin. (B) Upregulation of Dppa5, CyraB, Cdh3, and Sox2 following 5-aza-2-deoxycytidine treatment. Closer examination of the expression analysis indicated that the genes, displayed in Figure S4A, become upregulated following 5-Aza-2-deoxycytidine treatment. The relative expression ratios of Dppa5, Alpha crystallin B, and P-cadherin are similar to that of sox-2, suggesting that sox-2 may play a role in the regulation of these genes. Expression values extracted from normalized microarray data. (0.09 MB TIF) [file pone.0008340.s010.tif]
